# Implicit and Explicit Voice Training Effects on Speech-on-Speech Perception and Listening Effort

**DOI:** 10.1097/AUD.0000000000001805

**Published:** 2026-03-11

**Authors:** Ada Biçer, Deniz Başkent, Carolyn McGettigan, Thomas Koelewijn

**Affiliations:** 1 Department of Otorhinolaryngology/Head and Neck Surgery, University Medical Center Groningen, University of Groningen, Groningen, the Netherlands; 2 Research School of Behavioral and Cognitive Neuroscience, Graduate School of Medical Sciences, University of Groningen, Groningen, the Netherlands; 3 Speech, Hearing and Phonetic Sciences, Division of Psychology and Language Sciences, UCL, London, United Kingdom.

**Keywords:** Explicit voice training, Implicit voice training, Listening effort, Speech intelligibility, Voice familiarity, Voice training

## Abstract

**Objectives:**

In multitalker listening situations, intelligibility of the target speech improves when spoken by a personally familiar voice or with trained voices. Yet, the literature is inconclusive on which voice training method, implicit or explicit, is most effective. The objective of the present study was to systematically evaluate implicit and explicit training benefits on intelligibility and listening effort in a speech-on-speech listening task.

**Design:**

Of 32 normal hearing participants, half received implicit voice training via a speech intelligibility task, while the other half received explicit voice training via a speaker recognition task. For both training methods, the same stimuli, uttered by the same talkers, and the same number of sentences were used. Following voice training, all participants completed a speech-on-speech listening task. Stimuli consisted of a target and a simultaneously presented masker sentence, presented at −6, 0, and +6 dB target to masker ratios (TMRs), uttered by a trained and an untrained voice. During this task, in addition to speech intelligibility, pupillometry measurements were recorded to assess listening effort.

**Results:**

There was no significant effect of voice training on speech intelligibility, for either implicit or explicit voice training, and no significant difference in intelligibility scores between the two training methods. Nevertheless, increasing the TMR significantly improved speech intelligibility. A significant interaction between voice training method and TMR was shown, and post hoc analysis revealed better speech-on-speech listening performance after implicit than explicit voice training at +6 dB TMR. Time-based analysis of the pupillometry data showed smaller pupil dilation responses (less effort) for both implicit and explicit voice training groups, in some TMR conditions. For trained voices only, pupil dilations were significantly smaller for explicitly trained voices than implicitly trained voices in the −6 and +6 dB TMR conditions.

**Conclusions:**

Results from the present study demonstrated that both implicit and explicit voice training might lead to a reduction in listening effort, even in the absence of a speech intelligibility benefit. Overall, listening to voices that were explicitly trained seemed to be less effortful than listening to implicitly trained voices. Nevertheless, implicit voice training might be more beneficial in improving speech intelligibility performance for more favorable TMRs. Therefore, both implicit and explicit voice training might provide differential benefits for speech-on-speech perception. Nevertheless, voice training effects reported in the present study can reflect a combination of stimulus-specific perceptual learning and voice familiarity, as the same stimuli were used during training and testing, with generalization to novel utterances left for future studies.

## INTRODUCTION

Familiarity with voices, such as those of partners and friends, may improve intelligibility in a speech-on-speech listening situation (Johnsrude et al. 2013; Holmes et al. 2018; Domingo et al. 2020). This can happen implicitly, through exposure to voices in daily life, and can improve intelligibility even when people are unaware that they were listening to someone familiar (Holmes et al. 2018). Becoming familiar with a voice can also happen explicitly, when speaker identity is known to the listener (Newman & Evers 2007). While gaining voice familiarity is a continuous occurrence in daily life listening, its effect can also be investigated experimentally, by means of voice training.

Implicit voice training strongly resembles how listeners become familiar with voices in real life, making it ecologically valid (Sidtis & Kreiman 2012; Lee & Perrachione 2022). An implicit voice training paradigm can involve listening to an audiobook (McKenzie et al. 2021; Biçer et al. 2023), performing a speech intelligibility task that is not related to the speaker’s identity (Kreitewolf et al. 2017), or exposure to a voice while playing a video game (Flaherty et al. 2024). In contrast, in an explicit voice training paradigm, the task involves learning the talker’s identity and their associated voice, in general by means of a speaker recognition task. In the initial training phase of this task, names and associated voices are presented to the participants, while in the execution phase listeners are asked to identify the name of the talker who utters the speech (Nygaard et al. 1994; Nygaard & Pisoni 1998; Yonan & Sommers 2000; Holmes et al. 2021) or make old/new talker judgments (Lavan et al. 2019).

While most studies focus on one type of voice training method only, Yonan and Sommers (2000) assessed the effect of both implicit and explicit training in the course of two experiments. The outcomes showed a main effect of voice training on speech intelligibility, which did not significantly differ between the implicit and explicit voice training groups. Newman and Evers (2007) showed that explicitly knowing a talker identity results in better performance in a speech shadowing task, compared with listening to a familiar voice whose identity was not disclosed, and therefore only implicitly known (Newman & Evers 2007). However, there are also studies where implicit voice training shows no performance benefit (Case et al. 2018). Overall, it is important to systematically compare implicit and explicit voice training methods to better understand the distinct learning mechanisms implicit and explicit voice training rely on.

By examining in more detail what makes listeners recognize talkers, Lee and Perrachione (2022) showed that the ability to identify talkers was greater after explicit voice training, but even implicit voice training led to an improvement in explicit talker recognition scores. It is important to note that, in this study, the distinction between implicit and explicit voice training was achieved by manipulating the degree of attentional focus on the talker’s vocal characteristics. These results suggest that differences in attentional allocation during voice training can affect how efficiently talker information is represented and accessed during subsequent speech perception. While implicit voice training relies on exposure and the involvement of automatic attention mechanisms, explicit voice training involves task-relevant learning of talkers voices, and it requires directed attention (Lee & Perrachione 2022). Since both voice training approaches might lead to behavioral benefits, it is especially important to assess if cognitive demands of speech perception differ between these voice training methods, given that the underlying cognitive (attention) mechanisms might be different.

While cognitive aspects of listening might be potentially different following these voice training methods, previous research indicates that, in general, processing familiar voices might be cognitively less demanding than processing unfamiliar ones. Maibauer et al. (2014) assessed reaction times during a word recognition task including famous and nonfamous talkers and found that word recognition was faster for famous talkers. Authors argue that talker-specific voice cues for familiar voices affect word recognition at a relatively early processing stage. This suggests that early voice cue perception is cognitively less demanding for familiar voices compared with unfamiliar voices, perhaps because the talker normalization process is cognitively less demanding for familiar voices. Talker normalization refers to the assumption that to deal with talker differences, a real-time mapping of voice characteristics and acoustic-phonetic categories is needed (Nusbaum & Morin 1992; Magnuson et al. 2021). While talker normalization assumes that variability across talkers should be actively normalized, exemplar-based or episodic models suggest that listeners may store memory traces of speech, including acoustic characteristics of talkers, which could lead to better speech recognition (Goldinger 1996). More recently, an ideal adapter framework was proposed providing a combination between talker normalization and episodic models, and the authors suggest that listeners dynamically adjust their expectations for a specific talker as they gain experience (Kleinschmidt & Jaeger 2015). Taken together, the above-mentioned studies imply that listening to familiar voices might be cognitively less demanding, either by reducing the cognitive demands of talker normalization processes, by using stored memory traces, or through adaptive learning.

Listening to speech can be cognitively demanding for normal hearing listeners in cocktail-party situations, such as with talkers or noise in the background (Zekveld et al. 2010; Koelewijn et al. 2012; Mattys et al. 2012; Wendt et al. 2018; Książek et al. 2021). The cognitive load exerted during listening—often referred to as *listening effort*—can be measured, for instance, by means of dual-task paradigms where a reduction in secondary task performance indicates increased effort (Kahneman 1973; Gagné et al. 2017), or by assessing changes in the pupil diameter by means of pupillometry, where an increase in the pupil diameter reflects an increase in cognitive load (Kahneman & Beatty 1966). Listening effort is used as an umbrella term and it represents a combination of attention, arousal, motivation, and listener, as well as source-related factors during listening (Pichora-Fuller et al. 2016). While listening effort represents directed attention, it can also reflect automatic attention, such as responses to novel or sudden (e.g., surprising) stimuli, as indicated by pupillometry studies (Preuschoff et al. 2011; Gomes et al. 2021; Graves et al. 2021). Related to that, larger pupil dilations were observed with stimulus uncertainty, even when listeners were not aware of the ambiguity in the stimuli, suggesting the involvement of implicit mechanisms or perhaps automatic attention (Graves et al. 2021). By means of pupillometry, it has been shown that talker variability in a speech-on-speech task affected the baseline pupil size and the mean pupil diameter (Koelewijn et al. 2015), which could indicate that talker uncertainty may affect listening effort.

In a recent study, we looked into the effect of a short-term implicit voice training on voice cue discrimination and listening effort with nonprocessed and vocoder-degraded speech (Biçer et al. 2023). We showed that listening effort was reduced for trained voices compared with untrained voices, but only for vocoder-degraded speech. It is interesting to note that there was no benefit of voice training on voice discrimination performance for both nonprocessed and vocoder-degraded speech. Therefore, as a first step, we wanted to assess the potentially differential effects of implicit and explicit voice training methods on speech intelligibility and listening effort during a speech-on-speech listening task, using nonprocessed speech heard by normal hearing listeners. Comparing the listening effort spent during implicit and explicit voice training provides insight into which voice training method might lead to a more efficient use of cognitive resources during voice analysis (e.g., talker normalization) conducted during speech perception.

### Present Study

In this study, we investigated how different voice training methods, implicit or explicit, affect speech-on-speech intelligibility and listening effort with normal hearing listeners. Implicit voice training consisted of a speech intelligibility task and explicit voice training consisted of a speaker recognition task. Both tasks used the same speech stimuli uttered by the same talkers, and the same number of sentences were used, which was 400 for the trained voice. However, it is important to note that there is no consensus in the literature on the duration of voice training and whether it differs between implicit and explicit voice training paradigms. While voice training benefit on speech intelligibility might improve with longer duration of voice training, Holmes et al. (2021) suggests that 88 sentences were sufficient to improve speech intelligibility in an explicit voice training paradigm. Similarly, previous research shows that implicit voice training that lasted around 30 minutes can reduce listening effort for trained voices (McKenzie et al. 2021; Biçer et al. 2023). Therefore, the aim of the present study was to assess the effects of short-term voice training, so that the potential clinical applicability of such a voice training paradigm for hearing-impaired listeners can be evaluated.

Following either voice training method, speech intelligibility was assessed, using an adaptation of the Coordinate Response Measure (CRM) test (Bolia et al. 2000; Brungart 2001). In previous studies, closed-set intelligibility tests such as CRM test were used to assess the effect of voice familiarity and voice training (Johnsrude et al. 2013; Kreitewolf et al. 2017; Holmes et al. 2018, 2021; Domingo et al. 2020), showing an improvement in speech perception with familiar or trained voices. To maximize a possible voice training effect, the same short sentences were used for the voice training and the CRM intelligibility test (Nygaard et al. 1994; Yonan & Sommers 2000; Newman & Evers 2007). The same type of stimuli was used during training and testing as it was indicated that training and testing with different types of materials, such as training with sentences and testing with words would require linguistic generalization, which may not be evident in voice training paradigms (Nygaard & Pisoni 1998). Similarly, training with sentences and testing with consonant–vowel (CV) triplets did not lead to a voice training benefit in speech discrimination (Biçer et al. 2023).

During the CRM intelligibility test, the target speech, which was either from a trained voice or an untrained voice, was presented simultaneously with masker speech at different target to masker ratios (TMRs), while at the same time, participants’ pupil size was recorded. We expected to see a benefit of voice training by an increase in speech intelligibility performance and/or by a reduction in listening effort, compared with listening to untrained voices, presented as target speech. Therefore, the benefits that could be drawn from voice training can be examined in both intelligibility (behavioral performance) and cognitive load (pupil size) domains, as previous studies indicated the possibility that voice training may show a benefit by only reducing listening effort (such as in McKenzie et al. 2021; Biçer et al. 2023), by only improving speech intelligibility, or perhaps, most advantageously, by doing both (such as in Sommers et al. 2015). As voice familiarity might show the largest benefits when listening situations are not optimal (Nygaard & Pisoni 1998; Yonan & Sommers 2000; Souza et al. 2013), we expected an interaction between TMR and voice training. More specifically, we expected trained voices to show a benefit in either behavioral performance or listening effort domain when TMRs were more disadvantageous (such as at −6 and 0 dB TMR). We also expected speech intelligibility to increase and listening effort to decrease at +6 dB TMR.

## MATERIALS AND METHODS

### Participants

Thirty-six adults participated in the study, of which 4 participants were excluded due to insufficient audiometry or quality of pupillometry data. Therefore, results from 32 participants were included in the analysis. The ages of participants were in the range of 19 and 53 (mean age = 25, median age = 23, SD = 6.48), while the self-reported gender indicated participation from 10 men, 21 women, and 1 transgender individual. The choice for the participant number was based on a previous study conducted in our lab looking into the effect of voice training using pupillometry (Biçer et al. 2023). In addition, a simulation-based power analysis, using Monte Carlo simulations implemented in the “*simr”* package (Green & MacLeod 2016), based on the analysis model structure and α = 0.05, revealed that 32 participants in total yielded enough power (80% and above) to capture large (*d* = 0.8) and medium size main effects (*d* = 0.5), as defined by Cohen’s *d* (Cohen 1988). However, it should be noted that the small effects (*d* = 0.2) and the interaction (for both medium and small effect sizes) are not reliably captured with the present sample size, and the results should be interpreted with caution. All participants were native Dutch speakers and had normal hearing, with hearing thresholds at or below 20 dB HL at octave frequencies between 250 and 4000 Hz. They had normal or corrected-to-normal vision, and no participant reported any neurological disorder. Additional demographics show that 9 participants had an HBO education (higher vocational education), and the remaining 23 participants completed a WO education (university-level education), according to the Dutch education system. One of the participants learned 1 foreign language (in addition to Dutch), whereas 16 participants reported having learned 2 foreign languages, 10 participants reported 3 foreign languages, and 5 participants reported 4 foreign languages. Of all participants, 1 was bilingual, and 4 spoke a regional dialect (Frisian). Twenty participants reported having played musical instruments, of which 15 received a formal music education before the age of 10.

Written informed consent was obtained from the participants at the start of the experiment and an hourly compensation of 8 euros was awarded for participation in the study. The study was approved by the institute’s Ethics Committee.

### Stimuli

In the present study, the Dutch CRM corpus was used, which consists of recordings of full sentences (Nagels et al. 2021). These sentences started with a dog or a cat call sign, followed by a color and number (e.g., “Laat de hond zien waar de blauwe vijf is” meaning “Show the dog where the blue five is”). In total, the corpus consisted of 56 sentences per call sign (dog or cat) for each speaker, as each sentence mentioned 1 of 7 colors (blue, black, brown, red, yellow, green, white; all monosyllabic words in Dutch) and 1 of 8 numbers (1, 2, 3, 4, 5, 6, 8, 10; with 7 and 9 left out as they are bisyllabic words in Dutch. Therefore, the probability of giving a correct response (when both the color and the number were selected correctly) due to chance was 1.79%. In addition to the original CRM corpus, which was recorded previously with 1 female speaker (Nagels et al. 2021), 3 new male and 2 new female speakers were recorded for the present study. All 6 speakers were native Dutch speakers (3 female and 3 male) without any nonstandard regional accent or dialect.

During both implicit and explicit voice training, each trial contained one target speaker. The target speaker was selected randomly from a group of either 3 male or 3 female speakers. It is important to note that half of the participants received voice training only with male voices and the other half only with female voices. During training, participants listened to either all three male voices or all three female voices, while one voice among them was presented more often than the other two, which was later used as the trained voice at test. The target sentence was chosen randomly from the set of 56 sentences available for each speaker, and the same sentence could be repeated in consecutive trials. A speech-shaped noise masker, which started simultaneously with the target sentences and continued for 250 msec after the target sentence was completed, was presented at a +6 dB TMR. This speech-shaped noise was created separately for masking the female and male target talkers, using the average long-term spectrum of the 3 combined female and 3 combined male voices, respectively. Stimuli were presented in speech-shaped noise to promote learning by keeping the task difficulty optimal for learning, that is, relatively easy so the participants could do the task, but challenging enough for them to stay engaged. In addition, intelligibility benefit from voice training was not significantly different between training in babble noise or training in quiet (Holmes et al. 2021), indicating that in the present study, training in noise would not introduce detrimental effects on voice training, compared with training in quiet.

During the CRM intelligibility test, which followed either voice training method, participants listened to sentences starting with a dog call sign followed by a color and number while ignoring a masker sentence. Similar to voice training, both target and masker sentences were chosen randomly from a closed set of 56 sentences, meaning that the same sentence, either for target or masker, or both, could be selected multiple times over the course of the test and repeated in consecutive trials. Although the call sign for the target and masker sentences were different (dog versus cat), it was possible for both sentences to contain the same color-number pair in a given trial. Target sentences were presented from the trained or untrained voice that could be either female or male. On each trial, a masker sentence starting with a cat call sign was presented simultaneously. In the present study, a single-talker masker sentence was selected, as it was shown that maskers that are linguistically similar to the target might lead to a voice familiarity benefit, while maskers in another language or unintelligible noise may not have the same effect (Holmes & Johnsrude 2020). While target and masker sentences were uttered by the same talker, voices used as the masker were adapted by shifting the fundamental frequency (F0) and vocal-tract length (VTL) together (F0 + VTL) using STRAIGHT software (Kawahara & Irino 2005), to systematically control the differences between the target and masker voices. For masking of the female target, the F0 + VTL of the female target talker’s voice was shifted toward a male voice (Gaudrain & Başkent 2015), between the range of −2 and −4 semitones (st) for F0, in steps of 0.5 st. and VTL was shifted between the range of +0.60 and +1.20 st, in steps of 0.15 st. Within this range, masker voices were selected randomly, on a trial-by-trial basis. For masking of the male target, F0 + VTL of the male target talker’s voice was shifted toward a female voice, using the same range with opposite plus and minus signs for F0 + VTL, as female voices are higher in F0, and female speakers tend to have shorter VTLs. In addition, the minimum st values for the F0(∓2)+VTL(±0.6) manipulation range were selected based on the just-noticeable difference (JND) scores of normal hearing listeners (Gaudrain & Başkent 2015), so that the target and masker voice differences could be detected by the participants. When measured from a target male voice towards a female reference voice, the average JND was 1.71 st combined F0 + VTL for normal hearing listeners, which corresponds to 1.63 st F0 and 0.52 st VTL (Gaudrain & Başkent 2015); therefore, slightly larger values for F0 and VTL manipulations were selected to ensure detectability for the minimum F0 + VTL shifts. Similarly, 1.71 st voice difference between the target and masker voices can lead to significant benefits in speech-on-speech listening for normal hearing listeners, with all participants scoring above chance level (Huet et al. 2022). Moreover, 4 st was selected as the maximum of the range for target and masker voice differences, for the experiment to remain challenging enough, as larger F0 + VTL differences between the target and masker voices would lead the two voices to be easily distinguishable both acoustically and perceptually. Each target voice (trained and untrained voices) was presented at three different TMRs of −6, 0, and +6 dB.

### Setup

For voice recordings, an NT1-A RØDE microphone with pop-shield (RØDE Microphones LLC, Silverwater, Australia), Audient Evo 4 USB audio interface (Audient Ltd., Hampshire, UK), and Adobe Audition software (Adobe Inc., California, USA) were used. Recordings were done at 24 bits and 44.1 kHz in an anechoic room. Sound levels of the stimuli were root-mean-square (RMS) equalized, and presentation level was later calibrated to 60 dB SPL, using Sennheiser HD 280 Pro headphones (Sennheiser GmbH & Co., Wedemark, Germany) and KEMAR Head & Torso simulator (GRAS Sound & Vibration, Holte, Denmark).

During the experiment, the auditory stimuli were presented through Sennheiser HD 280 Pro headphones connected to an Audient Evo 4 audio interface. The visual stimuli were presented on a Philips Brilliance 240B screen. MATLAB was used to run the experiment. Pupillometry measurements were obtained via a Tobii Pro Fusion eye-tracker (Tobii Pro AB, Tobii Technology, Stockholm, Sweden), which was attached centered below the monitor. For pupillometry measurements, illumination of the room was arranged by using two LED light panels (Ledgo E268CII Bi-color). These panels, placed on stands right and left of the participants’ seating position, were facing down. Light color was fixed at white light (daylight) and the brightness of the light was adjusted during the light calibration procedure. The sitting position of the participants was arranged according to the optimal distance from the eye-tracker, according to the Tobii Pro Eye-Tracker Manager software position guide, which was 50 to 80 cm, and the sitting position of the participants was monitored throughout the pupillometry measurements.

### Procedure

At the start of the experiment, participants provided a written informed consent and completed a demographic information questionnaire and pure tone audiometry screening (Interacoustics AC40 Clinical Audiometer, Interacoustics, Middelfart, Denmark). Half of the participants received implicit voice training (16 participants), and the other half received explicit voice training (16 participants) (see Supplemental Digital Content, Figure S1, https://links.lww.com/EANDH/B857, which illustrates the voice training paradigms). Within each voice training group (implicit or explicit), half of the participants were trained with a female voice and the other half were trained with a male voice, to balance out for a possible intelligibility bias toward one of the voices. During data analysis, these data were pooled and only trained (male and female) versus untrained (male and female) conditions were assessed.

During the implicit voice training, participants completed a CRM task. Participants first became familiar with the test and the test interface by completing five trials, which were not included in the analysis. It is important to note that participants did not receive any specific instructions to learn the voices used during this task. During the CRM task, participants selected the color and number pair they heard in the target sentence. A matrix showing all possible color and number combinations was displayed on the screen for response selection throughout the voice training session. Feedback was given by enlarging the correct color and number pair, following the response. In total, 600 sentences were presented during training, uttered by 3 different talkers of the same gender (either 3 female or 3 male speakers). While the sentences and the talkers were chosen randomly for each trial, 400 sentences were uttered by 1 talker, and the other 2 talkers uttered 100 sentences each. The talker that uttered most sentences was the same talker (either female or male) for all participants and was used for target speech in the trained condition of the intelligibility task. Implicit voice training on average lasted around 1 hr and 15 min, where the longest presented voice lasted around 50 min, and the other 2 voices were presented around 12 minutes each.

During the explicit voice training, participants listened to the sentences from the CRM corpus, but instead of performing the CRM task, they completed a speaker recognition task (similar to the task used by Holmes et al. 2021). First, during the familiarization phase, 5 sentences from each of the 3 talkers of the same gender were presented in silence with no background talker. During this phase, one specific name was displayed on the screen, while 5 sentences uttered by the paired speaker were presented in sequence, and this was repeated for each speaker and name pairings. The names were chosen randomly from commonly used Dutch male (e.g., Daan, Jan, Thijs) and female names (e.g., Femke, Sophia, Leanne). Participants were instructed to learn the voices of the speakers and their associated names. Following the familiarization phase, participants were presented with three boxes, where each of the 3 male or 3 female names were displayed. Participants were asked to select the name of the speaker that uttered the sentence by a mouse click on the corresponding box. Feedback was given by the selected name box flickering green, indicating a correct response, or by an orange light flickering in the location of the correct box, indicating an incorrect response. It is important to note that during both implicit and explicit voice training, the same 3 female or the same 3 male voices were used. Similar to the implicit voice training, one of the speakers uttered 400 sentences, while the other 2 speakers uttered 100 sentences each (600 sentences in total). The most frequently used voice was the same for half of the participants who underwent explicit voice training (male or female voice), and half of the participants who performed the implicit voice training. This most frequently used voice was later used as the target voice during the CRM intelligibility test. The duration of the explicit voice training was around 1 hr and 15 min, similar to the duration of the implicit voice training.

Following the completion of either voice training, the CRM intelligibility test was performed, during which the effect of voice training on intelligibility and listening effort was assessed simultaneously (Supplemental Digital Content, Figure S2, https://links.lww.com/EANDH/B858, which illustrates the CRM intelligibility test paradigm). First, the illuminance level for pupillometry was set by adjusting the brightness settings of the LED light panels until the average pupil diameter size was halfway the range as measured between the light (≈950 lx) and dark (<3 lx) illuminance conditions (Voltcrat LX-1108 light meter). In addition, a 5-dot location calibration was performed using the Tobii Pro Eye-Tracker Manager software.

Calibration was followed by the CRM intelligibility test, which was similar to the procedure of Nagels et al. (2021). The CRM intelligibility test consisted of 180 trials in total, where in half of the experimental runs, the target sentences were uttered by the most frequently used target voice in training (trained voice), and the other half of the target sentences were uttered by an unfamiliar voice (untrained voice). The untrained voice was either a female or a male voice, but always belonged to the opposite gender of the trained voice. Consequently, for half of the participants who received voice training with female voices, a male voice was untrained, and for participants who received voice training with male voices, a female voice was untrained. Therefore, two speakers were used as the target during the CRM intelligibility test, and while a target voice was trained for half of the participants, the same voice was an untrained voice for the other half of the participants. Each trial started with 3 sec of silence followed by the target and masker sentence, with a duration of 2.6 to 3 sec, followed by 2 sec of silence. During this part of a trial, which lasted around 8 sec, a white fixation dot was presented in the center of the screen, and the pupil diameter and the x and y gaze data of both eyes were recorded. After this time period, the CRM matrix interface containing all the colors and numbers appeared on the screen allowing participants to respond with a mouse click. No feedback was given after the response. Trained and untrained voices were presented at −6, 0, and +6 dB TMRs, resulting in a total of 6 conditions presented as blocks with each block containing 30 trials, while the order of the blocks was randomized for each participant. Using a blocked design avoids introducing task-unrelated variance to the pupillometry outcomes. In addition, it was previously shown that presenting stimuli in blocked or interleaved fashion did not have a significant effect on voice training benefit (Kreitewolf et al. 2017).

### Data Analysis

#### Speech Intelligibility Data Analysis

The analysis of the CRM intelligibility test results was in line with Nagels et al. (2021). Only trials where both color and number keywords were correctly selected were considered a correct response. The differences between receiving voice training with female or male voices were not assessed, but rather listening to trained versus untrained voices is assessed, regardless of the gender of the trained voice. The accuracy data from the CRM intelligibility test were analyzed with a Generalized Linear Mixed Model (GLMM) analysis, using R software (v4.0.2; R Core Team 2020), and the lme4 package (Bates et al. 2015). GLMM analysis allows us to perform analysis on non-normally distributed data, such as the present dataset, because of the ceiling performance of participants at +6 dB TMR. In addition, GLMM takes into account effects of random factors in the data, such as F0 + VTL differences between the target and masker voices and participants. A mixed-effects logistic regression model was fitted using the function *glmer* from the lme4 package. Furthermore, the analysis of variance (ANOVA) χ^2^ test was used to assess the best model fit. The lowest Akaike’s Information Criterion value was used as a measure in selecting the best fitting model (Akaike 1974). The best-fitting model had the following syntax: correct responses ~ voice training method*tmr + voice training + (1 |F0+VTL) + (1 + voice training |participant), where participant and F0 + VTL were included as random intercepts, while a random slope was fitted for voice training. The fixed effect structure was selected by a backward stepwise model comparison (Nagels et al. 2021). The full model which included a full interaction between all independent variables (e.g., voice training method*tmr*voice training) revealed that there were no significant three-way interactions, nor any two-way interactions involving voice training, which led to the chosen best-fitted model’s fixed effects structure. Random effects structure was selected by a forward model, by keeping the fixed effects structure intact. The random effects were started with (1 | participant). While a by-participant slope for voice training (1 + voice training | participant) improved the fit significantly, as was shown by a significantly lower Akaike’s Information Criterion and, other modes with random slopes fitted for F0 + VTL did not converge. Therefore, the best fitting model considers the change in baseline values of participants due to how sensitive they might be to voice training effects and accounts for systematic differences in masker voice manipulations. A logit link was used for the analysis of binomial data. A Type II Wald *χ*^2^ analysis was performed, using the car package (v3.1.2, Fox & Weisberg 2019), to evaluate the overall effects (Harding et al. 2023). The post hoc analysis was conducted using the emmeans package (v1.8.9, (Lenth 2023) ) and corrections for multiple comparisons were done with false discovery rate.

#### Pupillometry Data Analysis

Pupil diameter was recorded for both eyes at a sampling frequency of 120 Hz. Pupillometry data were first preprocessed using MATLAB, in line with the procedure described by Biçer et al. (2023). For each participant, the recordings of the one eye with the most valid data points were selected to be used in the analysis. Zero values in the pupil diameter traces from 1 sec before and 6.5 sec after the beginning of stimuli presentation were defined as eye-blinks. Trials that had 20% or more eye-blinks were removed from further analysis (5.83% of all trials of the study) and the remaining eye-blinks were corrected with linear interpolation, which was conducted from 10 samples before till 16 samples after the blink. In case eye movements were outside of the display area, these trials were removed from the dataset (2.67% of all trials). High-frequency artifacts were removed by applying an 11-point moving average smoothing filter. The remaining traces were baseline corrected by calculating the average dilation over a 1 sec time window before each trial, after which this value was subtracted from all the samples in that trace. The preprocessed pupil data were then averaged for each condition and participant.

First, pupillometry data were analyzed with more traditional measures by calculating the peak pupil dilation (PPD) and the average baseline. PPD calculations were made within the time window starting from the beginning of the stimulus presentation until 4.5 sec later, which was before response selection. PPD was used to assess the differences between conditions at the maximum value of the pupil dilations (in mm), relative to baseline. While PPD was an event-related measure, the averaged baseline was assessed as a metric that was not event-related, that represents cognitive load before the start of the trial. The average baseline was calculated for each participant and each condition, by averaging over the baseline values as collected for each trial before baseline correction. The first analysis was conducted as a 2 × 3 × 2 mixed ANOVA, with R software (v4.0.2; R Core Team 2020), using the ez package (v4.4.0; Lawrence 2016). Voice training (trained, untrained) and TMRs (−6, 0, +6 dB) were within-subject factors and voice training method (explicit, implicit) was included as a between-subject factor. Two separate ANOVAs were conducted for PPD and the averaged baseline as dependent variables.

The second analysis was a nonlinear regression, which was performed using the generalized additive mixed models (GAMMs). See van Rij et al. (2019) for details on how to conduct GAMM analysis with the pupillometry data. With GAMM analysis, smooth functions are fitted to the data, allowing us to capture both linear and nonlinear relationships between the experimental conditions. In addition, changes in the pupil dilation were assessed over time, without assessing outcome measures such as the PPD responses for each condition. The GAMM analysis was performed in R, using the packages mgcv (v1.8-31; Wood 2017) and itsadug (v2.4; van Rij et al. 2020). Pupillometry data were first preprocessed, and the eye movements and blinks were corrected, and the data were baseline corrected, as described earlier. Data were additionally down-sampled from 120 to 30 Hz and the application of the moving average smoothing filter was omitted from the preprocessing in order not to introduce additional autocorrelation between succeeding data points. The effects of voice training (trained, untrained) and TMR (−6, 0, and +6 dB) on the pupil dilation over time were assessed for implicit voice training and explicit voice training separately. Contrasts between trained and untrained voices in each TMR condition were assessed. Further, contrasts between implicitly trained voices and explicitly trained voices in each TMR condition were assessed. In addition to visualization methods, significances were tested for all three GAMM analysis by assessing the differences between levels of factors with a binary model, modeling intercept and nonlinear differences and with an ordered factor model, modeling intercept and nonlinear differences separately (Wieling 2018). With a binary model and an ordered factor model, difference smooths were fitted to assess the trained and untrained voice contrasts for each TMR condition, for implicit and explicit voice training groups separately. In addition, difference smooths were used to assess changes between implicitly trained and explicitly trained voices in each TMR condition.

For all three datasets, the following syntax was used for the initial GAMM: Pupil ~ Condition + s (Time, by = Condition) + s (Subject, bs = “re”) + s (Time, Event, bs = “re”), the *bam* function from the mgcv package was used and the heavily-tailed data was fitted using a scaled *t* distribution by using the *family =* “*scat”* argument (Wood et al. 2016). A random intercept was added for subjects, while a random slope was added for “Event.” Event represents a unique combination of Subject and Item (each trial). Random effects are indicated by “bs = re,” where “bs” denotes the basis function type and “re” indicates a random effect structure. More complex random effects (such as fitting random smooths for subjects and/or Items or including Event as a random intercept) were not added due to nonconvergence, and nonsignificant *p* values were shown for random effects, indicating that the more complex random effects were not necessary to explain the random structure in the data. In addition, comparison of the present GAMM model with models including more complex random effects was performed by using *compareML* function from itsadug package, and results revealed that there were no significant improvements in the model fit when more complex random effect structures were included. Further, *acf_resid* function from the itsadug package was used to control the autocorrelation errors. The output, representing autocorrelation at lag 1, was then added to the model described above. For the individual voice training method groups (implicit or explicit), “Condition” was defined as a factor of voice training and TMR conditions and estimated effects were calculated for each condition on the dependent variable “Pupil.” Addition of smooth terms is indicated by s(), while by = Condition refers to fitting of separate smooths per condition. For the GAMM analysis that looked at contrasts between the voice training methods, “Condition” was defined as a factor of voice training, TMR, and voice training method.

## RESULTS

### Voice Training Results

Mean percentage correct responses from implicit and explicit voice training were calculated, mostly to ensure that participants were attentive during voice training. The percent correct responses from the implicit voice training were high on average (M = 99.51%, SD = 3.66%), and no participant scored lower than 97.5% correct. Percent correct responses from the speaker recognition task performed during the explicit voice training were high on average (M = 98.57%, SD = 4.83%), and no participant scored lower than 97% correct.

### CRM Intelligibility Test Results

**Fig. 1.**
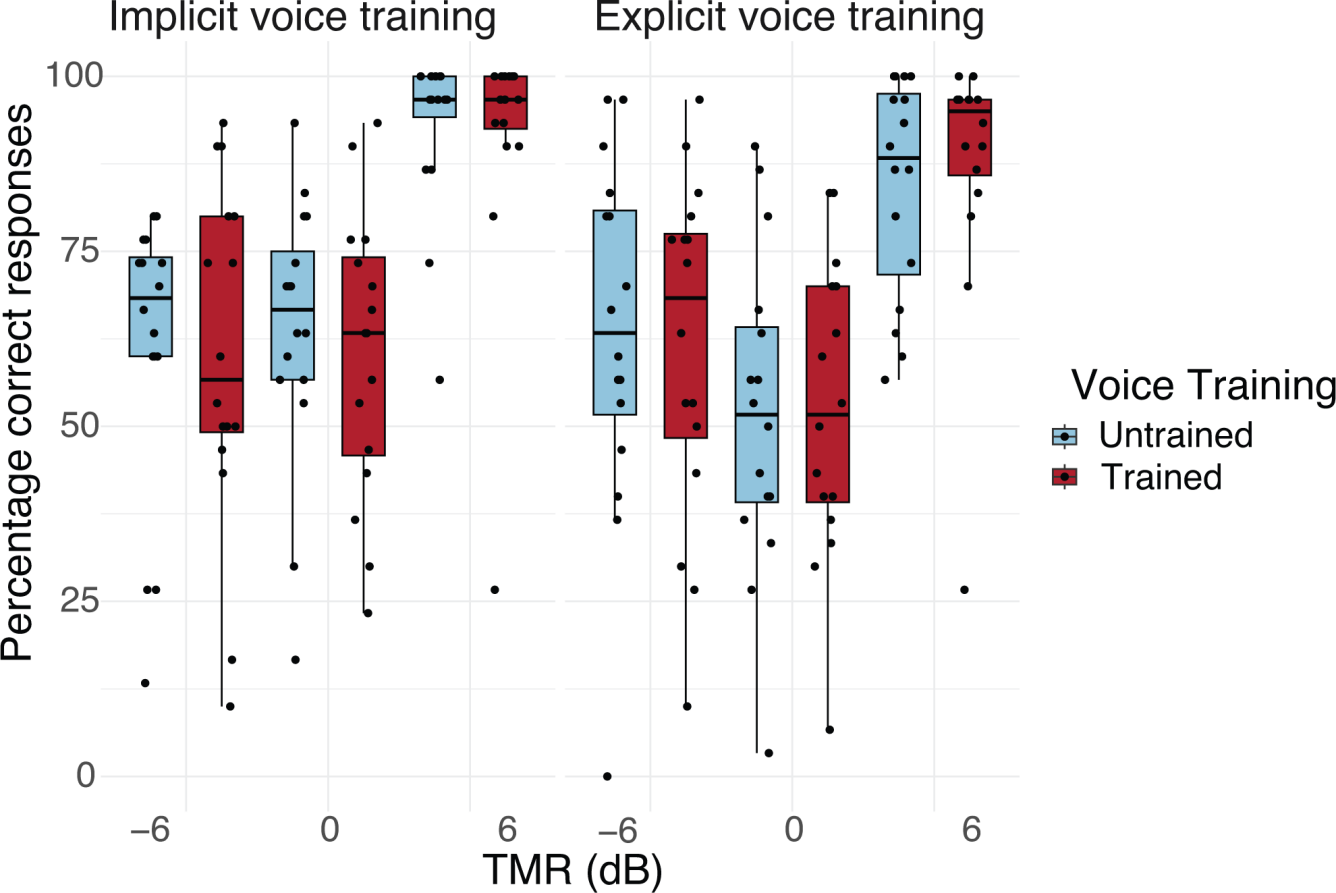
Results from the CRM intelligibility test are shown. Percentage correct scores are plotted for the 3 TMR conditions (−6, 0, and +6 dB), for untrained voices (blue color boxes) and for the trained voices (red color boxes). The first panel shows results from the implicit voice training group and the second panel shows results from the explicit voice training group. The boxes illustrate the upper and lower quartiles, the midline of the boxes represents the median, the whiskers indicate the highest and lowest values within 1.5 times the interquartile range, and the black dots represent individual participants’ data points. CRM indicates Coordinate Response Measure; TMR, target to masker ratio.

The outcomes of the CRM intelligibility test in the form of percentage correct scores are shown in Figure 1 as a function of TMR, for trained and untrained voices, and per voice training method group.

Results from the Type II Wald *χ*^2^ analysis showed that there was no significant main effect of voice training method (*χ*^2^(1) = 1.05, *p* = 0.31), nor voice training (*χ*^2^(1) = 0.01, *p* = 0.93). However, TMR had a significant main effect on the accuracy scores (*χ*^2^(2) = 508.73, *p* < 0.001). A significant two-way interaction between TMR and voice training method was observed (*χ*^2^(2) = 21.90, *p* < 0.001) and interaction contrasts are assessed with post hoc pairwise comparisons. There were no other significant two- or three-way interactions.

**Fig. 2.**
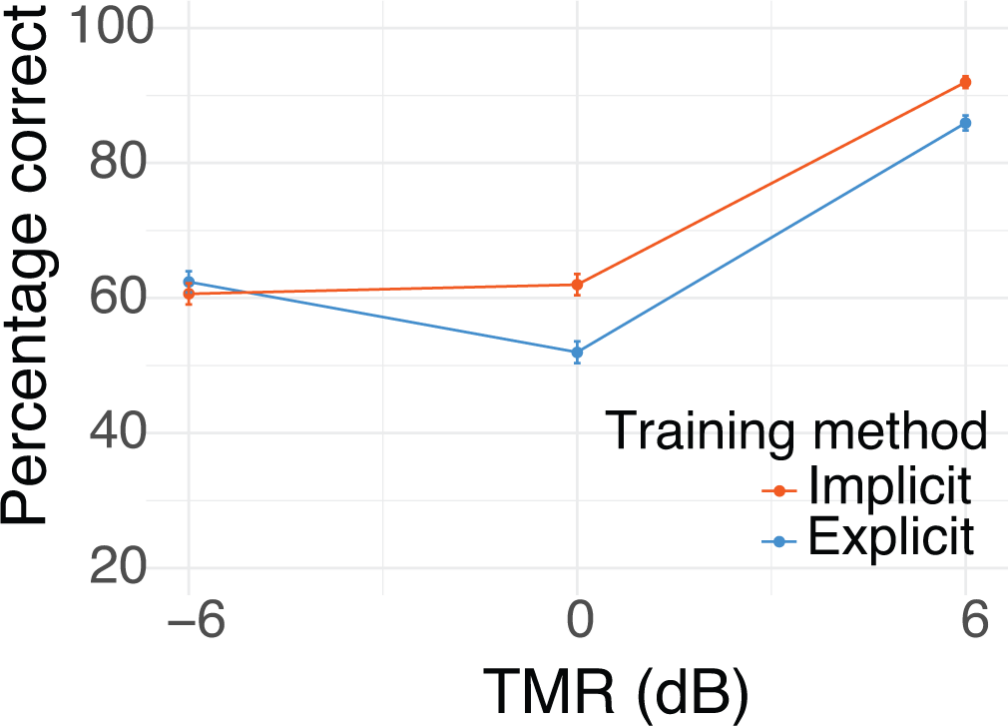
Interaction between TMR and voice training method is shown. Percentage correct responses are plotted as a function of TMR conditions, for implicit and explicit voice training methods, shown by red and blue lines, respectively. The error bars represent SE. TMR indicates target to masker ratio.

The two-way interaction between TMR and voice training method was assessed, and odds ratios (OR), SE, and *z* score values are reported. Results from the post hoc pairwise comparisons showed that the probability of a correct response was significantly different for explicit compared with implicit voice training when TMR was +6 dB (OR = 0.54, SE = 0.16, *z* = −2.05, *p* < 0.05), indicating a reduction in accuracy scores for explicit voice training group, compared with implicit voice training group at +6 dB TMR (Fig. 2). There were no significant differences between the explicit and implicit voice training groups at −6 dB TMR (OR = 1.09, SE = 0.30, *z* = 0.32, *p* = 0.75) nor at 0 dB TMR (OR = 0.62, SE = 0.17, *z* = −1.77, *p* = 0.08).

Furthermore, for implicit voice training group, increasing TMR from −6 to 0 dB TMR did not lead to a significant increase in accuracy scores (OR = 0.93, SE = 0.09, *z* = −0.70, *p* = 0.49), while increasing TMR from −6 dB TMR to +6 dB TMR (OR = 0.11, SE = 0.02, *z* = −15.49, *p* < 0.001) and 0 dB TMR to +6 dB TMR (OR = 0.12, SE = 0.02, *z* = −14.99, *p* < 0.001) led to significantly higher accuracy scores. For the explicit voice training group, while increasing the TMR from −6 to +6 dB (OR = 0.22 SE = 0.03, *z* = −12.26, *p* < 0.001) and from 0 to +6 dB (OR = 0.14, SE = 0.02, *z* = −16.31, *p* < 0.001) led to significantly higher accuracy scores, increasing TMR from −6 to 0 dB had the opposite effect. Results showed that accuracy scores were significantly lower when TMR was increased from −6 to 0 dB (OR = 1.64, SE = 0.17, *z* = 4.96, *p* < 0.001).

Finally, simple effects indicated that increasing TMR from −6 to +6 dB (OR = 0.16, SE = 0.02, *z* = −19.67, *p* < 0.001) and from 0 to +6 dB (OR = 0.13, SE = 0.01, *z* = −21.93, *p* < 0.001) increased accuracy scores significantly, while increasing TMR from −6 to 0 dB led to a significant drop in accuracy scores (OR = 1.24, SE = 0.09, *z* = 3.03, *p* < 0.01).

### Pupillometry ANOVA Results

Results from the 2 × 3 × 2 mixed ANOVA showed that only TMR had a significant main effect on the PPD [*F*_(2,60)_ = 15.22, *p* < 0.001]. Neither voice training [*F*_(1,30)_ = 1.99, *p* = 0.17], nor the voice training method significantly affected the PPD [*F*_(1,30)_ = 1.41, *p* = 0.24]. There were no significant interactions between the experimental conditions on the PPD (Supplemental Digital Content, Figure S3, https://links.lww.com/EANDH/B859, which illustrates the PPD ANOVA results). The ANOVA results on the dependent variable averaged baseline indicated that there were no significant main effects or interactions (Supplemental Digital Content, Figure S4, https://links.lww.com/EANDH/B860, which illustrates the descriptive statistics for PPD and averaged baseline results).

For speech-on-speech perception, the changes in the masker level do not always lead to monotonic changes in the intelligibility of the target speech. For example, around 0 dB TMR, intelligibility could be lower than even less favorable TMRs, due to lack of level cues that can help segregate speech and masker (Darwin et al. 2003). Based on this, pairwise comparisons between the levels of TMR were planned. These comparisons indicated that PPDs were significantly different between TMRs of −6 and 0 dB (*t*_(60)_ = −4.62, *p* < 0.001) and between TMRs of 0 and +6 dB (*t*_(60)_ = 4.92, *p* < 0.001), indicating that PPD was significantly larger at 0 dB TMR than at −6 and +6 dB TMR. There was no significant difference between PPDs, between TRMs of −6 and +6 dB (*t*_(60)_ = 0.31, *p* = 0.76).

### Pupillometry GAMM Analysis Results

**Fig. 3.**
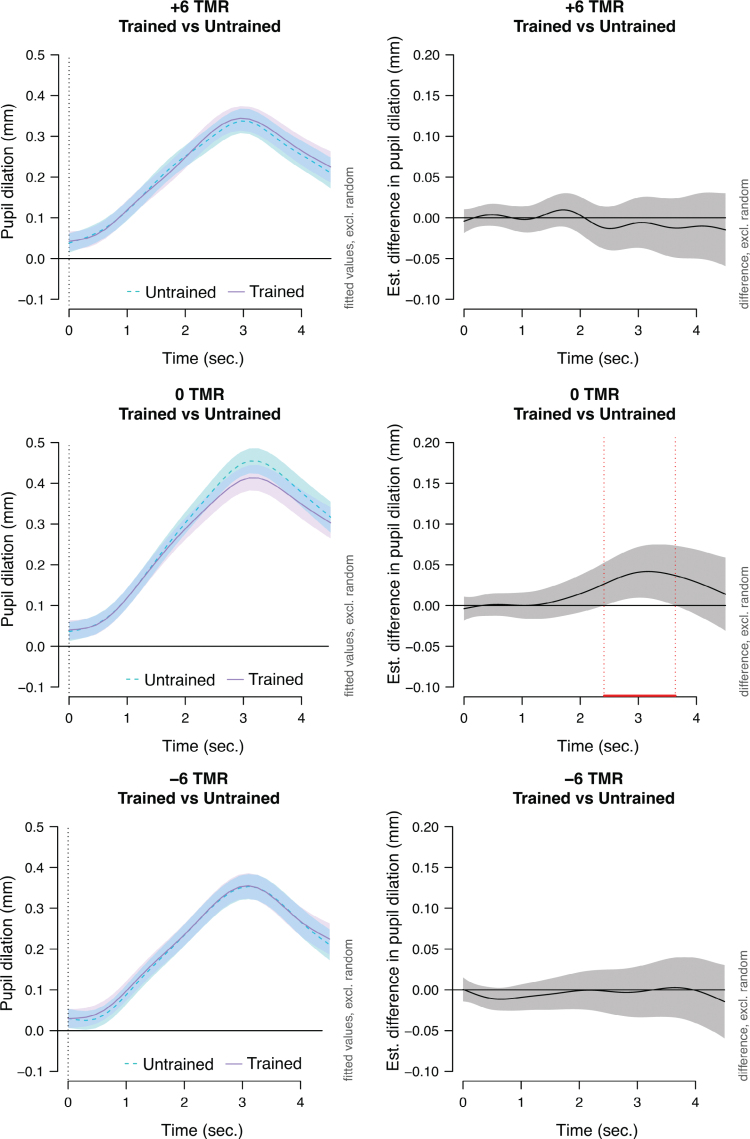
GAMM analysis results of the implicit voice training group. Stimuli were presented from 0 sec until approximately 2.6 to 3 sec, which was followed by 2 sec of silence. The plot shows the pupil dilation responses for the contrasts, and the estimated differences of pupil dilation to untrained and trained voices, for each TMR condition, over time, with pointwise 95% confidence intervals. Time periods where the estimated differences in pupil dilation between trained and untrained voices significantly differ from the 0 line are illustrated by a red line on the *x* axis of the estimated differences plots. Dashed lines illustrate the untrained voices and solid lines represent the trained voices. GAMM indicates generalized additive mixed model; TMR indicates target to masker ratio.

**Fig. 4.**
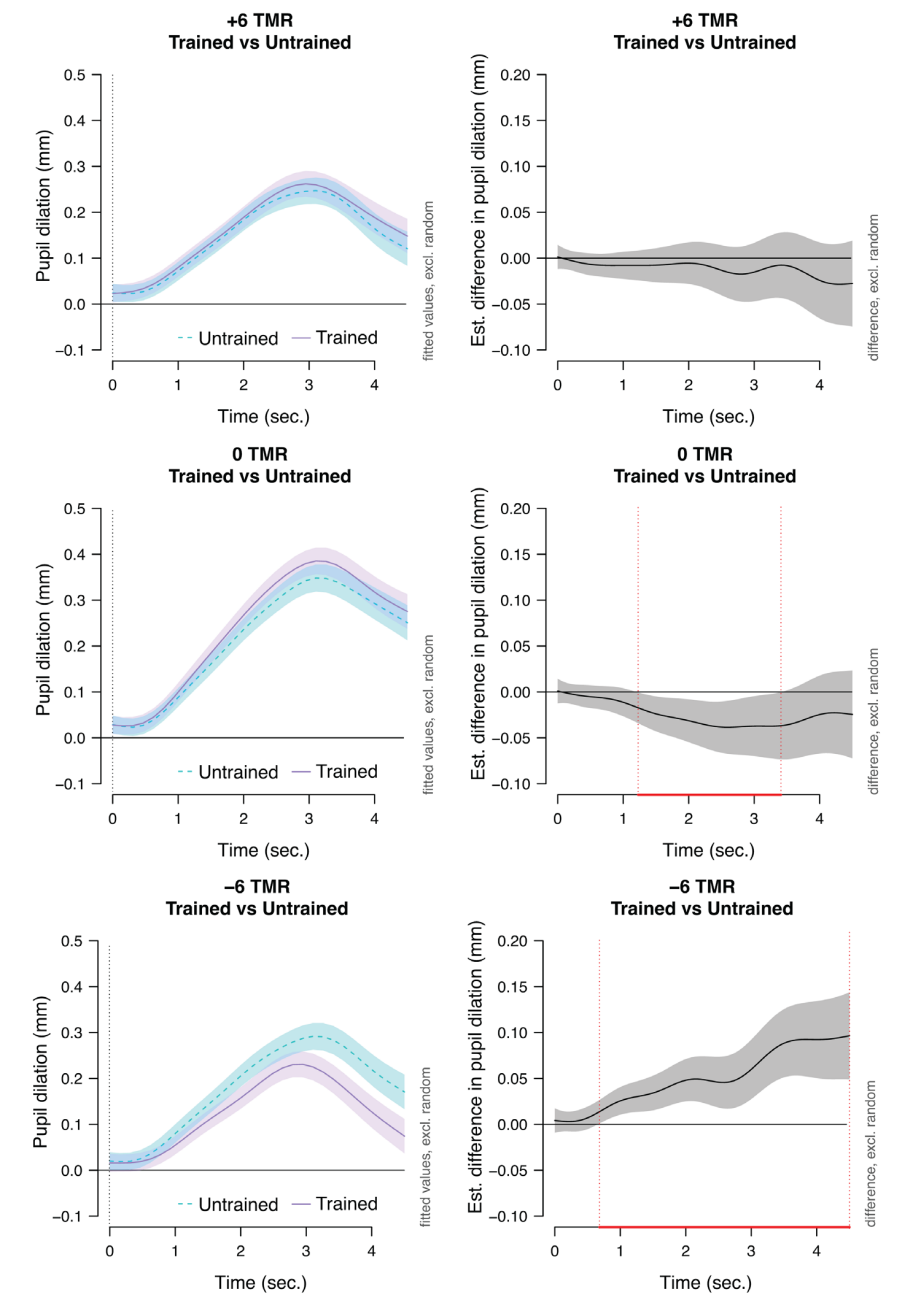
GAMM analysis results of explicit voice training group. Time periods where the estimated differences in pupil dilation between trained and untrained voices significantly differ from the 0 line are illustrated by a red line on the *x* axis of the estimated differences plots. Dashed lines illustrate the untrained voices and solid lines represent the trained voices. GAMM indicates generalized additive mixed model; TMR indicates target to masker ratio.

**Fig. 5.**
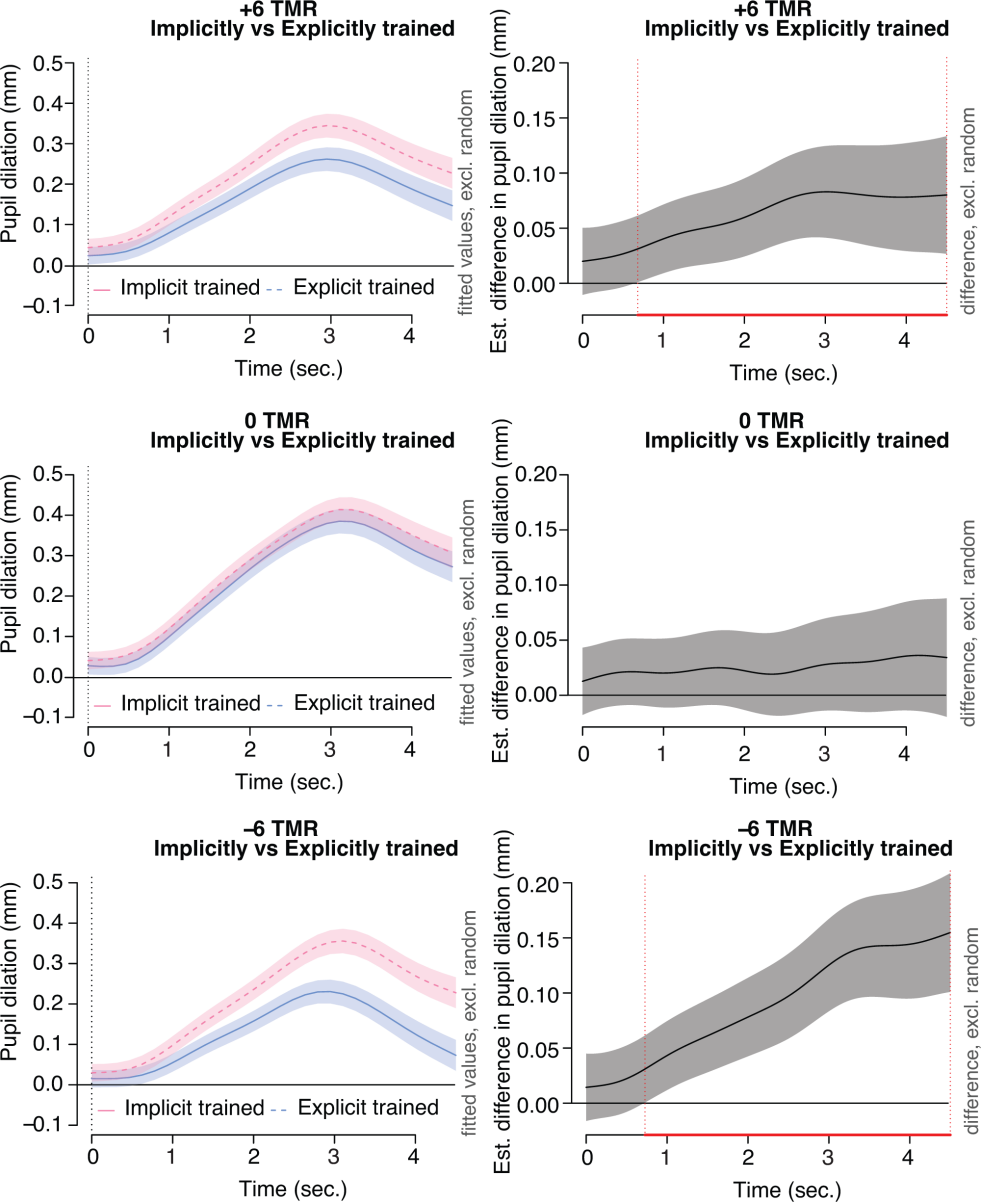
GAMM analysis results of the explicit and implicit voice training data combined. Contrasts between trained voices, namely explicitly trained and implicitly trained voices are shown. The solid lines represent the explicitly trained voices and dashed lines illustrate implicitly trained voices. Time periods where the estimated differences in pupil dilation between explicitly trained and implicitly trained voices significantly differ from the 0 line are illustrated by a red line on the *x* axis of the estimated differences plots. GAMM indicates generalized additive mixed model; TMR indicates target to masker ratio.

Pupil dilation responses over time were analyzed with GAMMs (in line with Biçer et al. 2023). In the present study, three different GAMM analyses were conducted. Figure 3 shows the estimated differences in the pupil dilation between untrained and trained voices for −6, 0, and +6 dB TMR, for the implicit voice training group, and Figure 4 shows the same for the explicit voice training group. On the right panel of the estimated difference plots, the significant deviation from the 0 line and thus the time period when the contrasted conditions significantly differ from one another, is illustrated with a red line. Similarly, Figure 5 shows the estimated differences in the pupil dilation and the time period where explicitly and implicitly trained voices significantly deviate, for each TMR condition. Significances between the experimental conditions were assessed with a binary model and an ordered factor model to assess the individual contribution of nonlinearity and its contribution together with intercept (linear) effects. Table 1 shows the summary statistics from the binary model for the implicit voice training group, while Table 2 shows the binary model results for the explicit voice training group. Table 3 shows the summary statistics of the binary model for the combined dataset, where data from the implicit and explicit voice training groups were merged, and only pupil responses of trained voices, explicitly trained and implicitly trained, were assessed. While results from the binary model are presented in Tables 1–3, results from the ordered factor model are explained below.

**TABLE 1.** Summary statistics from the binary model for implicit voice training group

Approximate Sig. of Smooth Terms	edf	Ref.df	*F*	*p* Value
s(Time): −6 dB TMR	8.66	8.97	352.61	<2e-16***
s(Time): 0 dB TMR	8.57	8.92	220.84	<2e-16***
s(Time): +6 dB TMR	8.51	8.94	296.62	<2e-16***
s(Time): Isminus6untrained	2.01	2.02	0.24	0.79
s(Time): Iszerountrained	4.91	5.99	2.87	< 0.01**
s(Time): Isplus6untrained	2.02	2.03	0.20	0.82
s(Subject)	14.73	16.00	2002.20	<2e-16***
s(Time, Event)	2614.22	2753.00	286.49	<2e-16***

The first column of the parametric coefficients represents the condition names. Edf, Ref.df, *F,* and *p* values of each condition, and their respective significance are shown. The Ref.df represents the reference number of degrees of freedom used, and edf value denotes the number of effective degrees of freedom. Significances are denoted by the *p* values. The condition names starting with “s(Time)” and followed by “Is,” represents the three difference smooths, showing the contrasts between trained and untrained voices for each TMR condition, for −6, 0, and +6 dB, respectively. Deviance explained = 63.2%.

Significance codes: *p < 0.05, **p < 0.01, ***p < 0.001.

TMR, target to masker ratio.

**TABLE 2.** Summary statistics from binary model for explicit voice training group

Approximate Sig. of Smooth Terms	edf	Ref.df	*F*	*p* Value
s(Time): −6 dB TMR	8.50	8.94	340.78	<2e-16***
s(Time): 0 dB TMR	8.63	8.96	287.08	<2e-16***
s(Time): +6 dB TMR	8.45	8.93	308.89	<2e-16***
s(Time): Isminus6untrained	2.00	2.01	9.15	< 0.001***
s(Time): Iszerountrained	3.87	4.64	4.17	< 0.01**
s(Time): Isplus6untrained	2.01	2.02	0.45	0.64
s(Subject)	14.70	15.00	3566.80	<2e-16***
s(Time, Event)	2602.18	2682.00	804.03	<2e-16***

Deviance explained = 63.1%.

Significance codes: *p < 0.05, **p < 0.01, ***p < 0.001.

TMR, target to masker ratio.

**TABLE 3.** Summary statistics from the binary model for the dataset of explicit and implicit voice training groups combined

Approximate Sig. of Smooth Terms	edf	Ref.df	*F*	*p* Value
s(Time): −6 dB TMR.trained	8.56	8.94	185.92	<2e-16***
s(Time): 0 dB TMR.trained	8.63	8.97	403.84	<2e-16***
s(Time): +6 dB TMR.trained	8.42	8.91	161.85	<2e-16***
s(Time): −6 dB TMR.untrained	8.63	8.97	392.10	<2e-16***
s(Time): 0 dB TMR.untrained	8.62	8.97	461.80	<2e-16***
s(Time): +6 dB TMR.untrained	8.46	8.93	325.27	<2e-16***
s(Time): Isimplicitminus6	3.75	4.51	9.72	<2e-16***
s(Time): Isimplicitzero	2.02	2.03	0.95	0.39
s(Time): Isimplicitplus6	3.74	4.49	3.98	< 0.01**
s(Subject)	30.44	32.00	2852.73	<2e-16***
s(Time, Event)	5228.23	5435.00	544.74	<2e-16***

Deviance explained = 63.3%

Significance codes: *p < 0.05, **p < 0.01, ***p < 0.001.

TMR, target to masker ratio.

#### GAMM Outcomes Implicit Voice Training

For the implicit voice training group, similar to the results of binary model (Table 1), results from the ordered factor model show that nonlinear differences of pupil dilation responses were significantly different between the trained and untrained voices at 0 dB TMR [*F*_(3.91,4.99)_ *=* 3.43, *p* < 0.01] and not at −6 dB TMR [*F*_(1.00,1.01)_ *=* 0.00, *p* = 0.99] or at +6 dB TMR [*F*_(1.01,1.03)_ *=* 0.35, *p* = 0.55]. However, there were no significant intercept (constant) differences in pupil dilation between trained and untrained voices at 0 dB TMR (*t =* 1.61, *p* = 0.11), nor at −6 dB TMR (*t =* −0.35, *p* = 0.73) and +6 dB TMR (*t =* −0.45, *p* = 0.65). Therefore, the significant differences between pupil responses while listening to trained and untrained voices at 0 dB TMR are mostly explained by nonlinear patterns in the data. A visual inspection of the estimated difference plots indicates that pupil dilation responses were significantly larger for untrained voices than trained voices at 0 dB TMR (Fig. 3).

#### GAMM Outcomes Explicit Voice Training

For the explicit voice training group, results from the ordered factor model were similar to the binary model (Table 2), showing that the nonlinear differences in the pupil dilation were significantly different between the trained and untrained voices at −6 dB TMR [*F*_(1.01,1.01)_ *=* 16.82, *p* < 0.001] and at 0 dB TMR [*F*_(2.87,3.64)_ *=* 3.75, *p* < 0.01]. However, there were no significant differences between the trained and untrained voices at +6 dB TMR [*F*_(1.02,1.03)_ *=* 0.81, *p* = 0.36]. Intercept differences between trained and untrained voices revealed that pupil responses were significantly different at −6 dB TMR (*t =* 4.26, *p* < 0.001) and 0 dB TMR (*t =* −2.07, *p* < 0.05), while there were no significant intercept differences between the trained and untrained voices at +6 dB TMR (*t =* −0.95, *p* = 0.34). Visual inspection of the estimated difference plots indicates that at −6 dB TMR, pupil dilation was significantly larger while listening to untrained voices than trained voices. However, at 0 dB TMR, pupil dilation responses were larger while listening to trained voices than untrained voices (Fig. 4). The deviance explained from the ordered factor model for the explicit voice training group was 63.1% and the deviance explained from the implicit voice training group’s data was 63.2%.

#### GAMM Outcomes Implicit Versus Explicit Voice Training

For the combined dataset, results from the ordered factor model showed similar results to binary model (Table 3), indicating that non-linear differences in the pupil dilation responses were significantly different between the explicitly and implicitly trained voices at −6 dB TMR [*F*_(2.75,3.51)_ = 12.60, *p* < 0.001] and +6 dB TMR [*F*_(2.74,3.49)_ *=* 4.36, *p* < 0.01] while there were no significant differences in pupil responses at 0 dB TMR [*F*_(1.02,1.03)_ = 0.67, *p* = 0.41]. Similarly, intercept differences between the pupil dilations while listening to explicitly trained and implicitly trained voices were significantly different at −6 dB TMR (*t =* 5.60, *p* < 0.001) and +6 dB TMR (*t =* 3.23, *p* < 0.01) and not significantly different at 0 dB TMR (*t =* 0.24, *p* = 0.81). Visual inspection of Figure 5 indicates pupil dilation responses were significantly smaller while listening to explicitly trained voices, compared with implicitly trained voices at −6 and +6 dB TMR. The deviance explained from the ordered factor model of the combined dataset (explicit and implicit voice training group data combined) was 63.4%.

## DISCUSSION

The goal of the present study was to examine how implicit and explicit voice training affect speech-on-speech intelligibility and listening effort. The speech intelligibility performance results showed that there was no overall significant difference between the implicit and explicit voice training methods, which is in line with Yonan and Sommers (2000). In addition, for both voice training methods, no intelligibility benefit from voice training was observed, also in line with what is reported previously in the literature (Case et al. 2018; McKenzie et al. 2021; Kanber et al. 2022). Average pupil responses were assessed as a non–event-related measure, which could indicate anticipatory effects before the start of experimental conditions (Koelewijn et al. 2015). As expected, no significant effects were found on the baseline pupil responses. While voice training did not have a significant effect on the PPD, results from the GAMM analysis showed that, at 0 dB TMR, for participants who received implicit voice training, pupil dilation responses were smaller for trained voices compared with untrained voices, indicating reduced listening effort with voice training. Similarly, for the explicit voice training group, at −6 dB TMR, pupil dilation responses were smaller for trained voices, indicating reduced listening effort for trained voices than untrained voices. It is interesting to note that in the explicit voice training group, at 0 dB TMR, pupil dilation responses were larger for the trained voices than for untrained voices, indicating more listening effort for trained compared with untrained voices. Finally, when pupil dilation responses to explicitly and implicitly trained voices were assessed, results showed that pupil dilation responses were smaller for explicitly trained voices than implicitly trained voices, indicating less listening effort for explicitly trained voices than implicitly trained voices at −6 and +6 dB TMR.

### Behavioral Outcomes

While there is no consensus on the duration of voice training in the literature, it is shown that longer familiarity with voices could increase the speech intelligibility benefits from voice training (Nygaard et al. 1994; Kreitewolf et al. 2017; Holmes et al. 2021). However, long training sessions in turn might increase the overall fatigue in listeners, potentially confounding pupil responses (Pichora-Fuller et al. 2016), which we wanted to avoid in the present study. In addition, a short-term voice training was implemented in the present study as a preliminary step toward evaluating its potential clinical application with hearing-impaired listeners. For rehabilitation purposes, a short-term voice training is more feasible to implement, as dropout rates would be lower (Jiam et al. 2019).

The lack of voice training benefit on speech intelligibility performance should not relate to the type of stimuli used in the present study, as sentences have previously been shown to be effective materials to establish a voice training benefit on speech intelligibility (Nygaard & Pisoni 1998). Similar to the stimuli used in the present study, a closed-set keyword intelligibility test, including the CRM, was used in previous voice familiarity and voice training studies, where an improvement in intelligibility was observed (Johnsrude et al. 2013; Kreitewolf et al. 2017; Holmes et al. 2018, 2021). Nevertheless, using closed-set speech material during training might limit exposure to the talker’s voice spectrum. In addition, the CRM materials used in the present study might not provide ecologically valid listening situations due to limitations in linguistic meaning, which might have interfered with the voice training effect. Moreover, in the present study, the same target speech stimuli were used during both training and testing, and therefore, generalization was not required.

Masker voice F0 + VTL st differences were chosen to be slightly above the normal hearing threshold (Gaudrain & Başkent 2015), as voice training was shown to provide the most benefit in more difficult listening situations, like in our previous study when speech was spectrotemporally degraded through vocoding (Biçer et al. 2023). Nevertheless, the task in the present study might have been too challenging for some listeners, which might have interfered with the expected training effect. Some participants might especially have difficulties discriminating between the target and masker speech at the minimum F0 + VTL shift of 2.09 st, as this manipulation was slightly above the average discrimination threshold. Holmes et al. (2018) show that F0 and VTL can contribute to recognizing familiar voices and understanding them, but only when F0 and VTL manipulations were very large, and above threshold. Related to that, voice discrimination JNDs at the threshold level did not show any voice familiarity (Holmes & Johnsrude 2023) or voice training (Biçer et al. 2023) benefit. Therefore, very precise representations of F0 and VTL might not be most relevant for voice familiarity, even though F0 and VTL information, perhaps together with covarying voice cues, such as pitch contour, rate of speaking, intonation, and more, can contribute to identification of familiar talkers and intelligibility (Holmes et al. 2018; Lavan et al. 2021; Holmes & Johnsrude 2023). Related to that, the results from the present study overall show a large variation between individual participants, suggesting that listeners might opt for different strategies. For instance, some listeners might not benefit from small differences in F0 + VTL voice cues for a training benefit to emerge. Instead, some might make use of differences in other voice cues (in addition to F0 + VTL), which were kept identical between the target and masker voices in this study.

Changes in TMR level were considered to affect the task difficulty, and a voice training benefit was expected predominantly at lower TMR levels. The present results show that, even though there was a benefit of explicit voice training on listening effort at −6 dB TMR, and an effect of implicit voice training on listening effort at 0 dB TMR, these effects were observed in the absence of a speech-on-speech intelligibility benefit. This is in line with Kanber et al. (2022) who found no benefit of voice familiarity on CRM test performance for multitalker babble as a masker at −6 dB signal to noise ratio. They argue that the implemented task might have been too easy to provide a familiarity benefit on intelligibility, as performance was around 80% correct. Compared with Kanber et al., intelligibility results in the present study were lower, suggesting that the task was more difficult; the performance at −6 dB TMR was around 60% correct, perhaps due to using a single-talker masker compared with multitalker babble (Simpson & Cooke 2005). Even with increased task difficulty, the present study did not show any performance benefit. However, listening was less effortful for trained voices in specific conditions of explicit or implicit voice training groups. It is worth noting that Kanber et al. reported more accurate voice-identity judgements for personally familiar voices than lab-trained voices. Therefore, it is possible that compared with listening to personally familiar voices, lab-trained voices might not be as effective in improving voice-identity recognition, even though this effect might not reflect on intelligibility performance (Kanber et al. 2022).

The performance outcomes showed a significant main effect of TMR. As expected, speech intelligibility improved when TMR was +6 dB compared with −6 or 0 dB TMR. It is interesting to note that increasing the TMR from −6 to 0 dB had no effect on the intelligibility performance for implicit voice training, and even reduced intelligibility performance for the explicit voice training group. Previous research has shown similar outcomes (Darwin et al. 2003), indicating that the absence of level cues between the target and masker speech at 0 dB TMR can make speech segregation more challenging (Egan et al. 1954). The interaction between TMR and voice training method was further shown at +6 dB TMR, where intelligibility scores were significantly better for the implicit voice training group than for the explicit voice training group. It is worth mentioning that this effect might be related to perceptual learning of the task itself, as the same paradigm was used during both training and testing for the implicit voice training group, especially considering that trained voices were not more intelligible than untrained voices in any condition. In addition, the implicit voice training group might be susceptible to familiarity with the sentences more strongly than the explicit voice training group. This could be due to the directed attention toward the content of the sentences during both training and test for the implicit voice training group, while for the explicit voice training group, listeners’ attention was directed at voice differences during training. Therefore, the improvement in intelligibility at +6 dB TMR for the implicit voice training group compared with the explicit voice training group might partially reflect some confounding effects due to training and testing with the same materials and the use of the same experimental paradigm.

### Pupillometry Outcomes

Pupillometry results analyzed by means of an ANOVA showed no significant PPD differences between the trained and untrained voices and implicit and explicit voice training groups. There was, however, a main effect of TMR, which showed that for both implicit and explicit voice training, PPDs were larger at 0 dB TMR compared with −6 and +6 dB TMR, indicating an increase in listening effort. This finding coincides with the reduction in intelligibility scores at 0 dB TMR compared with −6 and +6 dB TMR, which further supports the idea that the reduction in performance at 0 dB was due to increased task difficulty.

In line with our previous study (Biçer et al. 2023), pupillometry results were analyzed with GAMMs in addition to the traditional pupillometry measures. In the present study, the target speech and the single-talker masker were presented simultaneously. While this design was chosen to maximize the voice familiarity benefit (Holmes & Johnsrude 2020), it raises the possibility that the early onset pupil responses might represent pupil responses to stimulus onset rather than experimental conditions (Winn et al. 2018). Therefore, assessing the time course of the pupil dilation responses with the GAMM analysis can overcome this potential confounding factor. In addition, the results from the GAMM analysis show that there were no significant differences between trained versus untrained voices in the first 0.5 sec of stimuli presentation, indicating that the results presented here are unlikely to represent pupil responses to stimulus onset, but instead represent event-related responses to experimental conditions. Analysis of pupillometry data from the implicit voice training group, using GAMMs, showed that at 0 dB TMR, pupil dilation was larger for untrained voices than trained voices, indicating less listening effort for trained voices. For the explicitly trained group, the same effect was shown at a TMR of −6 dB. This finding indicates that challenging listening conditions can show a benefit in reducing listening effort from voice training in normal hearing listeners. Unexpectedly, for the explicit voice training group, at 0 dB TMR, pupil dilation was larger for trained voices than untrained voices, suggesting that listening to untrained voices was less effortful than trained voices.

In the present study, the 0 dB TMR condition introduces a unique challenge, as a simultaneous single-talker masker was presented without intensity cues. This condition not only led to larger pupil dilation responses shown by PPD results of both voice training groups, but also the implicit voice training group showed relatively lower listening effort for trained voices. However, the explicit voice training group showed a different pattern of results, indicating that not only at 0 dB TMR CRM intelligibility performance dropped, but also listening to trained voices was more effortful. Taken together, in this unique condition, implicit voice training might be effective in reducing listening effort for trained voices and more resistant to challenges introduced by the lack of intensity cues during a speech intelligibility test. It might be possible that with implicit voice training, perception of trained voices is more resilient to voice cue competition introduced from the masker due to better perceptual learning related to performing the CRM task for a longer time period or due to a more sensitive perceptual tuning mechanism involved with passive exposure to trained voices during the implicit voice training. It might be possible that with explicit voice training, perceptual tuning required to distinguish between the target and masker voices was not as efficient, which might have led to an increase in listening effort during 0 dB TMR, when target and masker voices are more difficult to segregate.

As mentioned in the Framework for Understanding Effortful Listening (FUEL), listening effort reflects the involvement of many factors, including directed and automatic attention, motivation of the listener, fatigue, and speech-source related factors, among listener-related factors (Pichora-Fuller et al. 2016). Therefore, an alternative explanation for the increase in pupil responses while listening to trained voices at 0 dB TMR condition for the explicit voice training group might be related to the listener’s directed attention or motivation. More specifically, these results might reflect deployment of cognitive resources toward the talker that will lead to more intelligible speech perception in a challenging listening situation, which was, in this case, trained voices. Similarly, Mechtenberg et al. (2024) reported larger pupil responses while processing of clear speech compared with casual speech, contrary to their expectations. Authors suggests that additional cognitive resources were directed toward the clear speech signal, as it would lead to more accurate speech perception. Therefore, listeners in the explicit voice training group may have been more consciously aware of when they were listening to a trained voice compared with listeners in the implicit voice training group, especially considering that the trained voice was associated with a talker identity, such as a name. This could lead the listeners to strategically direct their attention to the trained voice, particularly under the most challenging experimental condition, as an attempt to compensate for the decrease in intelligibility. Nevertheless, this increase in cognitive processing led to an unsuccessful compensation, as intelligibility did not increase for trained voices at 0 dB TMR for the explicit voice training group.

Further GAMM analysis, focusing only on the trained voices, revealed that pupil responses were significantly smaller for explicitly trained voices than implicitly trained voices for the −6 and +6 dB TMRs conditions. However, at 0 dB TMR, there were no differences between the implicitly and explicitly trained voices. These results show that processing of explicitly trained voices was less effortful than implicitly trained voices when level cues were available. This finding is not surprising considering that during explicit voice training, the focus of the listener was on the voices of the talkers and the differences between voices, instead of the content of the speech, which would involve a separate mechanism to decode the semantic meaning. Therefore, as a short-term training method, explicit voice training might lead to less effortful listening.

In line with earlier studies (Nusbaum & Morin 1992; Zhang & Chen 2016), the present results suggest that talker normalization is cognitively less demanding once acoustic cues of voices are learned, as in the case of familiar voices (Maibauer et al. 2014). In other words, the decrease in listening effort for trained voices, compared with untrained voices, might originate from a reduction for the need of an elaborate mapping of voice characteristics through talker normalization. Alternatively, voice training might ease access to stored memory traces of speech or optimize adaptive learning mechanisms (Goldinger 1996; Kleinschmidt & Jaeger 2015), which might decrease the cognitive demands of listening. In the present study, the masker voices deviated from target voices by shifts of the F0 + VTL voice cues. Therefore, a strategy that might be used during the CRM intelligibility test involved differentiating F0 + VTL voice cues between the target and the masker for an intelligible speech perception. Voice training possibly led to better and cognitively less demanding retrieval and usage of talker-specific voice cues.

Our results, showing that listening to trained-to-be-familiar voices might not require as much cognitive effort during speech-on-speech perception compared with untrained voices, can be incorporated with the traditional model of voice-identity processing (Belin et al. 2004, 2011) and with the prototype model of voice-identity recognition (Lavner et al. 2001). These two models were recently combined (Maguinness et al. 2018), and identification of unfamiliar voices was incorporated into the combined model. According to the combined model of voice-identity recognition, recognition of unfamiliar voices would require a repetitive analysis of voice characteristics, as unfamiliar voices would not match any existing reference patterns. Through repetitive exposure to the same voice, these references can be established for the unfamiliar voices. This process of mapping new reference patterns, such as in talker normalization, would require deployment of additional cognitive resources. A recently introduced model called person perception from voices proposes incorporating person characteristics such as age, sex, or psychological state with the talker’s voice, during talker recognition from voices (Lavan & McGettigan 2023). According to the person perception from voices model, unfamiliar voices can contain information about the physical or psychological state of the talker, which can contribute to speaker recognition, even though the talker identity is not known. Our results suggest that perhaps listening to an unfamiliar person leads to the deployment of additional cognitive resources or perhaps direction of attentional mechanisms, to map the person characteristics information together with the new voice information, to achieve a successful person perception from voices. In the voice-identity processing models, recognition of familiar voices is mostly considered as recognizing familiar voices but not trained voices. As the training implemented in the present study was short-term compared with being familiar with voices in real life, it is interesting to see that even exposure to 400 sentences might lead to the establishment of new voice reference patterns or less effortful person perception from voices. Nevertheless, in the present experimental paradigm, the same speech corpus was used during both training and test. While this ensures that linguistic generalization would not be required, which could have hindered the voice training effect (Nygaard & Pisoni 1998; Biçer et al. 2023), exposure to the same speech corpus might have led to familiarity with the exact voice-stimuli association during training. This might have contributed to the observed voice training benefit at test. Therefore, in the present study, voice training benefit on listening effort might reflect a combination of stimuli- and voice familiarity, which should be considered when interpreting the findings.

Taken together, the present study showed that short-term voice training did not yield a robust intelligibility benefit, but it affected listening effort under specific conditions. Future study might assess if extending the duration of voice training and incorporating naturalistic speech materials that provide a wider variety of the talker’s vocal repertoire might improve voice training outcomes. In addition, voice training effect on intelligibility could be tested with larger F0 and VTL voice differences between the target and masker speech, as voice familiarity might not depend on very precise representations of F0 and VTL (Holmes & Johnsrude 2023). Furthermore, it would be informative to assess speech-on-speech intelligibility and listening effort when different speech corpora are used during training and testing. Evaluating performance with different speech corpora would indicate whether the voice training benefits observed in the present study are generalizable across different speech materials. Finally, voice training effects could be assessed in listeners with hearing loss, such as cochlear implant users. More specifically, future research could assess if voice training would lead to a decrease in listening effort in cochlear implant users, to assess if voice training benefits are comparable to normal hearing listeners in the present study. Therefore, future study would not only clarify the mixed effects of training observed in the present study, but it would also aid the development of optimized voice training protocols to improve real-life speech communication.

## CONCLUSION

Overall, neither the implicit nor the explicit voice training method showed a benefit in intelligibility scores, while the implicit voice training group had slightly better intelligibility scores than the explicit voice training group in +6 dB TMR condition. In addition, both implicit and explicit voice training led to a reduction in listening effort in some conditions. Furthermore, explicit voice training might lead to less effortful listening than implicit voice training. The mixed outcomes shown at the 0 dB TMR conditions warrant further investigation. Overall, the present study highlights the importance of using physiological metrics like pupillometry during speech perception tests to assess changes in cognitive load.

## ACKNOWLEDGMENTS

The authors thank Dr. Etienne Gaudrain for providing the experimental software and contributing to the data analysis, and Elif Gülgeç for helping with data collection. The authors also thank the Center for Information Technology of the University of Groningen for their support and for providing access to the Habrok high-performance computing cluster.

## Supplementary Material
