# Supplementary material for: Implicit and Explicit Voice Training Effects on Speech-on-Speech Perception and Listening Effort
Source: Ear Hear. 2026 Mar 11;47(4):1109–24. doi: 10.1097/AUD.0000000000001805 (PMC13252977; doi:10.1097/AUD.0000000000001805)
Supplement: Supplementary file 1 [file aud-47-1109-s001.pdf]

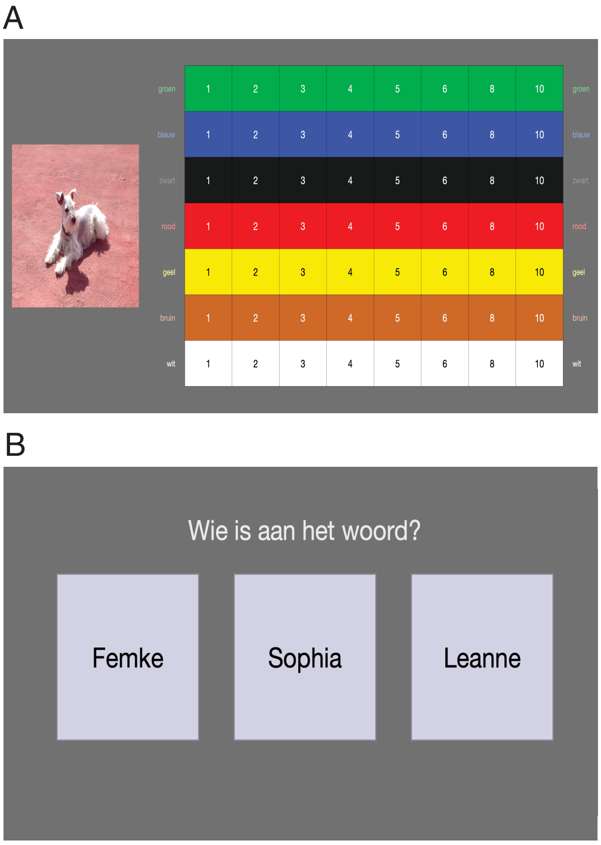

Figure S1. A. The CRM matrix interface, displayed during the implicit voice training. B. The speaker recognition task interface, displayed during the explicit voice training.
