# Supplementary material for: Implicit and Explicit Voice Training Effects on Speech-on-Speech Perception and Listening Effort
Source: Ear Hear. 2026 Mar 11;47(4):1109–24. doi: 10.1097/AUD.0000000000001805 (PMC13252977; doi:10.1097/AUD.0000000000001805)
Supplement: Supplementary file 2 [file aud-47-1109-s002.pdf]

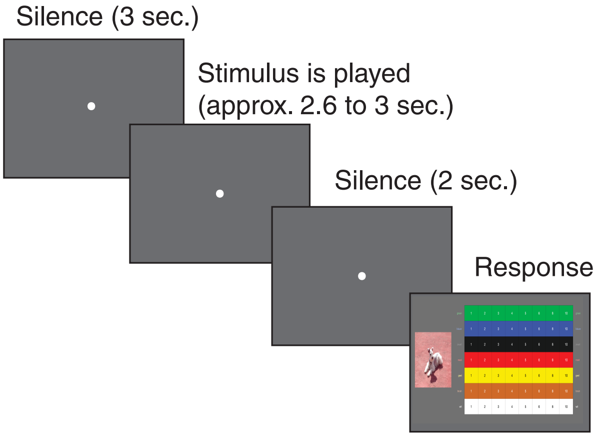

Figure S2. Illustration of one trial, displayed during the CRM intelligibility test. The trial started with 3 seconds of silence, followed by the stimulus presentation and another 2 seconds of silence. A white fixation dot was presented during this time, and pupillometry measurements were acquired. Following the 2 seconds of silence, the CRM matrix was displayed for response selection.
