# Supplementary material for: Implicit and Explicit Voice Training Effects on Speech-on-Speech Perception and Listening Effort
Source: Ear Hear. 2026 Mar 11;47(4):1109–24. doi: 10.1097/AUD.0000000000001805 (PMC13252977; doi:10.1097/AUD.0000000000001805)
Supplement: Supplementary file 3 [file aud-47-1109-s003.pdf]

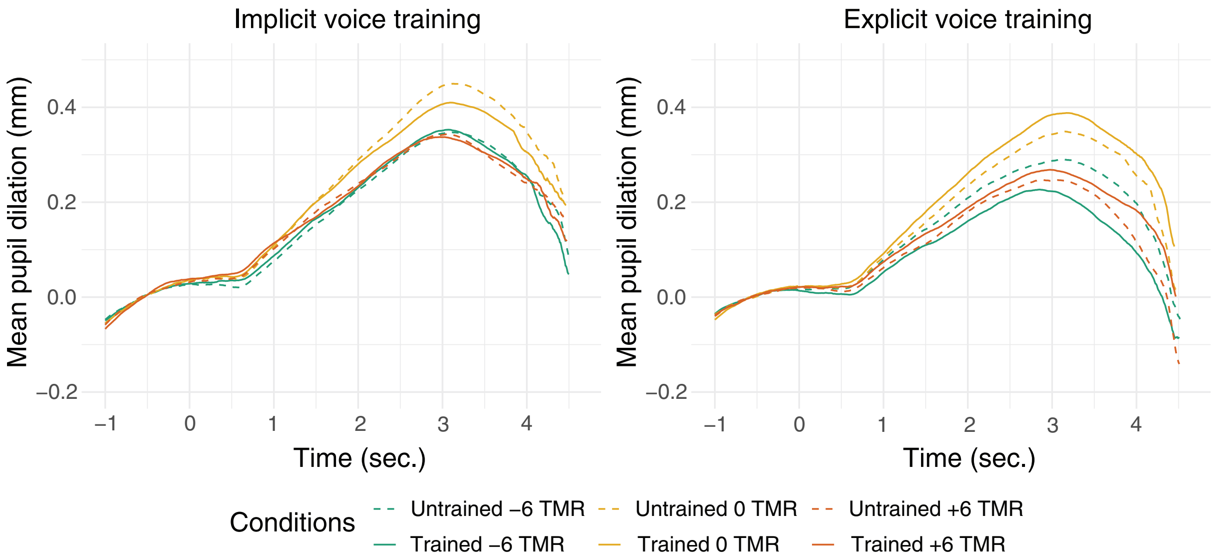

Figure S3. The mean pupil dilation (in mm) over time (in seconds) relative to the baseline for trained and untrained voices in different TMR conditions is shown. The left and right panels show results from implicit and explicit voice training groups, respectively. In both panels, the solid lines represent trained voices, and the dashed lines represent untrained voices. TMR conditions are color coded. The x-axis represents the time from 1 second prior to stimulus presentation (from -1 to 0 seconds), until the end of the last 2 seconds of silence within one trial.
