# Supplementary material for: Implicit and Explicit Voice Training Effects on Speech-on-Speech Perception and Listening Effort
Source: Ear Hear. 2026 Mar 11;47(4):1109–24. doi: 10.1097/AUD.0000000000001805 (PMC13252977; doi:10.1097/AUD.0000000000001805)
Supplement: Supplementary file 4 [file aud-47-1109-s004.pdf]

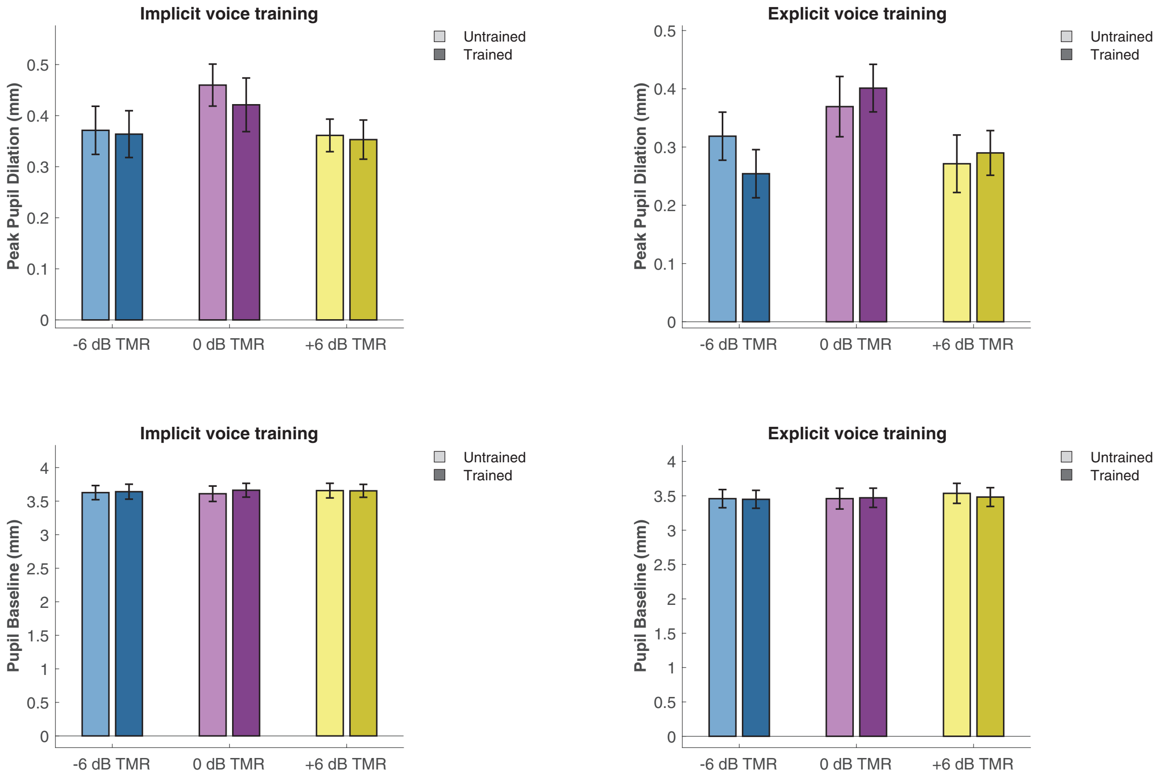

Figure S4. Descriptive statistics for the Peak Pupil Dilation (PPD) and the averaged baseline outcomes are shown in the top and bottom panels, respectively. The left panels show results from the implicit voice training group while the right panels show results from the explicit voice training group. Bar plots represent pupil responses to trained and untrained voices across the TMR conditions. Error bars represent the standard error of the mean.
